# Effect of optimized collagenase digestion on isolated and cultured nucleus pulposus cells in degenerated intervertebral discs: Erratum

**DOI:** 10.1097/MD.0000000000014142

**Published:** 2019-01-04

In the article, “Effect of optimized collagenase digestion on isolated and cultured nucleus pulposus cells in degenerated intervertebral discs”^[1]^, which appeared in Volume 97, Issue 44 of Medicine, the authors would like to note funding from “Shanghai Pudong New Area Health Planning Commission funding project (PW2015A_12).”
